# Construction and refined management of a pre-prescription review system: a real-world study in a tertiary hospital

**DOI:** 10.3389/fphar.2026.1791155

**Published:** 2026-03-26

**Authors:** Jiajia Gui, Lin Zhang, Dan Liu, Shan Li, Nan Mou, Ruoning Li, Yingying Xu, Jing Hu

**Affiliations:** Department of Pharmacy, The First Affiliated Hospital of Army Medical University, Chongqing, China

**Keywords:** pharmacist, pre-prescription review system, prescription and medical order review, refined management pathway, review efficiency and accuracy

## Abstract

**Objective:**

Information technology-driven pre-prescription review system (PPRS) is critical pillars for medication safety. How to balance the efficiency and accuracy of review has become a core issue. This study aims to retrospectively analyze the establishment and application effectiveness of the PPRS for rational drug use. It summarizes the system’s limitations and operational challenges encountered, further explores refined management pathways for the system, and provides insights and considerations for smart healthcare to assist clinical practice in promoting rational drug use.

**Method:**

A single-center real-world retrospective analysis study was conducted at a tertiary hospital in Chongqing, China. Using evidence-based methods, a descriptive analysis was conducted on the construction and refined management path of PPRS, and its effectiveness was evaluated. Before and after the PPRS went online, prescription and inpatient order data were monitored. Unpaired sample t-test and one-way ANOVA were used to study the primary outcome of the rationality rate of prescriptions and medical orders, and the secondary outcome of the types of unreasonable prescriptions and the changes in system warning levels.

**Results:**

The construction and refined management of PPRS have significantly increased the rationality rate between the total prescriptions (92.53% vs*.* 99.94%, P < 0.0001) and medical orders (97.77% vs*.* 99.99%, P < 0.0001). The proportion of prescriptions with high problem proportions decreased significantly after intervention, such as repeated medication (24.94% vs*.* 3.85%, P < 0.0001). In addition, following implementation, the proportion of prescriptions with usage and dosage issues (34.31% vs*.* 19.51%) also decreased before intervention. The number of PPRS intercepted alerts has increased annually, with the proportion of Level 3 prescription alerts (12.77% vs*.* 15.71%) and Level 4 medical orders alerts (42.40% vs*.* 55.48%) increased, while the proportion of Level 2 alerts for prescriptions (2.61% vs*.* 0.91%) and medical orders (2.12% vs*.* 1.04%) generally showed a downward trend, reducing the frequency of invalid alerts.

**Conclusion:**

The construction and implementation of PPRS is associated with enhancing the rationality of prescriptions and medical orders. Under the guidance of the refined management pathway, a replicable template has been established to support clinical practice in smart healthcare, reduce invalid alerts, and promote personalized medication.

## 1 Introduction

With the vigorous development of information technology and artificial intelligence (AI), intelligent decision support systems have demonstrated significant potential in ensuring patient medication safety, reducing drug-related problems (DRP) (Chustecki, 2024; Mennella et al., 2024), improving diagnostic accuracy, and optimizing treatment plans (Ghafur et al., 2020). As a leading cause of preventable harm in healthcare, DRP is the key clinical problem that these intelligent systems aim to address. Among these, medication errors and adverse drug events are particularly prevalent (Krähenbühl-Melcher et al., 2007; Schindler et al., 2021), causing over 40 billion US dollars in economic losses globally each year, imposing a huge financial burden on healthcare systems (Donaldson et al., 2017). Fortunately, the vast majority of DRP are preventable. At all stages of medication use, healthcare professionals (including doctors, nurses, pharmacists, and home caregivers) can effectively avoid related problems through timely intervention (Camacho et al., 2024). As key players in medication management, pharmacists play an increasingly important role in ensuring the safety, rationality and effectiveness of medication.

Under the multidisciplinary collaborative diagnosis and treatment model, pharmacist-led medication intervention significantly improves the detection rate of medication-related problems (Reinau et al., 2019; See et al., 2020), and help reduce the incidence of adverse drug events, decrease the readmission rate and related economic losses caused by DRP (Sheikh et al., 2021). Thus, enhancing the participation of pharmacists in prescription review and medical order management can help improve the overall level of medication safety guarantee and provide greater support for clinicians' work. Although pharmacists have already demonstrated significant value in reducing DRP, traditional pharmacist-led interventions remain insufficient to address growing clinical needs. Consequently, information technology-driven pre-prescription review system (PPRS) has been developed to assist pharmacists in their review processes. Previous studies have confirmed the system’s ability to promote rational drug use (Yang et al., 2025; Yue et al., 2025).

**FIGURE 1:**
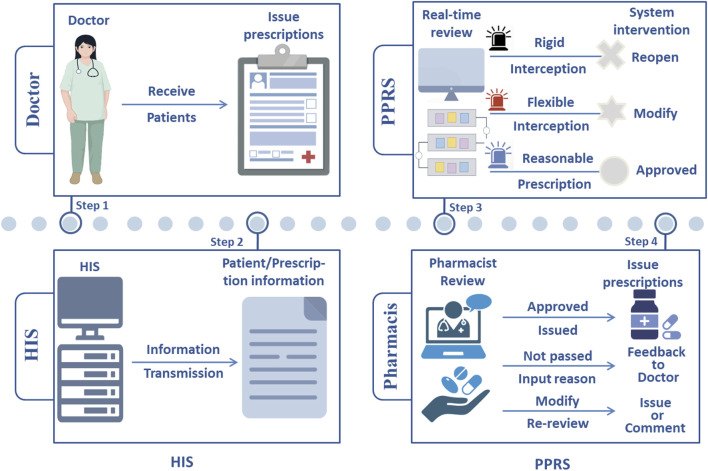
Schematic diagram of PPRS workflow. HIS: Hospital information system; PPRS: Pre-prescription review system.

However, there are still challenges in achieving effective pharmacist intervention within high-throughput pharmacy environment. Pharmacists in large general hospitals review a huge number of prescriptions daily, which can exceed 10,000. On average, they need to complete the review of one prescription every 30 s. In this high-intensity working condition, how to balance the review efficiency and accuracy has become a critical issue. While previous PPRS could assist pharmacists in improving review efficiency, their adaptability to complex clinical scenarios is limited, and they still suffer from issues such as insufficient accuracy and high false positive rates (Liu et al., 2021; Xie et al., 2022). An AI-assisted PPRS (Figure 1) can be viewed as a countermeasure. By integrating foundational data such as package inserts and specialized disease knowledge bases, this system enables automated preliminary reviews, thereby identifying potential medication risks in advance, reducing repetitive work for pharmacists, and enhancing the overall efficiency of prescription review. Meanwhile, adopting a dual-track approach combining “rigid” and “flexible” interception with parallel systematic screening and manual review can significantly reduce the rate of missed reviews, enhance its accuracy, and enable timely intervention during the pre-stages to minimize the occurrence of DRP.

This study aims to evaluate the establishment and application effectiveness of the PPRS through its real-world implementation, focusing on its role in reducing the rate of missed reviews, improving prescription compliance and accuracy rates, and summarizing the limitations and problems that occurred during the operation of the system. On this basis, the refined management pathway of the system was further explored, such as combining individualized inspection indicators and customizing prescription review rules with the help of flowcharts. These efforts aim to continuously optimize the prescription review process, promote personalized precision medication, minimize medication errors to ensure patient medication safety, thereby providing a replicable template for the implementation intelligent assistance systems in clinical practice and promoting the health practice of smart support systems in the healthcare field.

## 2 Methods

### 2.1 PPRS design and construction

This study was conducted at Southwest Hospital, a comprehensive tertiary grade-A hospital in Chongqing, China. The PPRS was designed and developed by Sichuan Youlian information technology Co., LTD. (Sichuan Province, China). PPRS uses the MySQL database. Based on the characteristics and demands of rational drug use in clinical practice, it integrates underlying data such as package inserts, guidelines, and online specialized disease knowledge bases (Up to date, Yao ZH), etc.

PPRS implements a hybrid data integration mechanism based on “event-driven as the primary approach, supplemented by periodic polling” to ensure the timeliness and zero loss of system alerts. Specifically, critical data (such as prescription issuance, medical order changes, etc.) is pushed in real-time to the PPRS (based on Apache Kafka, with an interval of less than 100 milliseconds), while non-critical data (such as drug directory updates, physician permission changes) utilizes an incremental polling mechanism every 5 min. Moreover, machine learning algorithms (Extreme Gradient Boosting XGBoost) are also integrated to build an intelligent core think tank (ICTT) embedded within the PPRS to evaluate the rationality of prescriptions and medical orders (Supplementary Figure S1). XGBoost randomly splits the retrospectively collected case data into a training set (70%) and a test set (30%) using a 7:3 ratio. It employs 5-fold cross-validation, where the training set is divided into five equal parts.Each iteration uses four parts for training and one part for validation, repeated five times to ensure the model’s generalization capability. The model performs hyperparameter tuning via Bayesian optimization and quantifies the contribution of input features to the prediction results using SHAP (Shapley Additive Explanations) values to enhance clinical credibility.

### 2.2 Management before and after PPRS online

A multi-disciplinary team (pharmacy department, clinical departments, information department, etc.) was established to communicate and cooperate with the system architecture engineers (Sichuan Youlian) to precisely identify clinical requirements. Then, standardized training was provided to all relevant clinical staff to ensure smooth operation and and use of PPRS. Additionally, prior to full system deployment, PPRS underwent a 2-week silent run, followed by a 1-month phased rollout across departments. During the initial month of full deployment, refresher training and on-site troubleshooting support (by system architecture engineers) were offered to ensure robust system operation.

### 2.3 System warning levels

PPRS classifies drug alert information into five levels based on the ICTT and the in-hospital medication situation (Figure 3). Among them, Level 1 background information (not displayed to doctors, no warning); Levels 2–3 represent attention and caution information (indicating potential medication risks, prompting doctors to review drug details and weigh the risks and benefits); Level 4 is not recommended information (indicating prescription errors that need to be modified, such as excessive dosage, repeated medication or severe interactions); Level 5 signifies contraindicated information (warning of obvious prescription errors, including drug allergies, prohibited administration routes, and contraindications in traditional Chinese medicine, etc.).

### 2.4 Construction and refined management of the rule bank

The PPRS rule bank has established over 2.6 million review rules based on ICTT, incorporating data from more than 221,000 package inserts, 23,000 clinical guidelines, 15,000 cases, and constantly updated machine learning rules, covering the vast majority of drug usage rules in our hospital. Based on hospital medication requirements and refined management approaches, 7043 custom review rules were developed, and this rule set is continuously updated.

### 2.5 Data collection

This study collected the data (pre-intervention data) monitored by the prescription automatic screening system (PASS) during the 6 months prior to the launch of the PPRS and the data of drug prescriptions and inpatient medical orders since the PPRS was implemented in November 2020. Among them, the relevant data of unreasonable prescriptions and medical orders were given special attention, evaluated and further analyzed.

### 2.6 Inclusion and exclusion

Different prescription review sections were launched at different times. The outpatient prescription review section began trial operation in November 2020, while the inpatient medical order review section started trial operation in May 2021. To ensure the stability of the data and eliminate the errors caused by other unmeasurable system operation factors during the early trial operation stage of the system, only the outpatient prescriptions from 2021 to 2024 and the inpatient medical order review data from 2022 to 2024 were included for further study.

### 2.7 Data analysis

All statistical analyses were conducted with GraphPad Prism 8.0 software. Descriptive analysis was conducted on the construction and refined management pathway of PPRS. Unpaired sample t-test was used to compare the changes in prescription and medical order rationality rates before and after the intervention, while One-way ANOVA was employed to analyze alterations in types of irrational prescriptions following the system’s implementation. P < 0.05 was considered statistically significant.

### 2.8 Study statement

To enhance the reporting quality of this study in the field of evidence-based practice for quality improvement and facilitate its comprehensibility and evaluation, the reporting of the present study was developed in accordance with the Evidence-Based Practice Quality Improvement (EBPQI) Reporting Guideline (Reynolds et al., 2026).

## 3 Results

### 3.1 Construction and operation of PPRS

**FIGURE 2:**
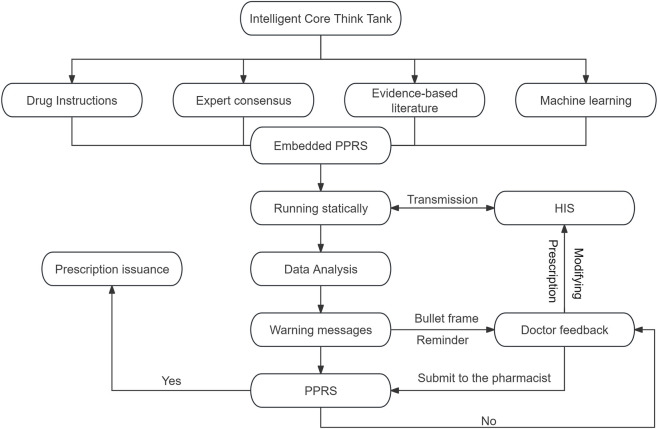
Flowchart of prescription review process. PPRS: Pre-prescription review system; HIS: Hospital information system.

PPRS was connected to the hospital information system (HIS) to achieve real-time data transmission. When a patient visits a doctor, the doctor issues a prescription and stores it in the HIS, and then the prescription information is transmitted to the PPRS for review. If the prescription is judged to be reasonable, the doctor can directly sign the prescription. If the prescription is judged to be unreasonable, the unreasonable information will be transmitted back to the doctor’s side of HIS from PPRS for warning, and the doctor can modify the information or submit it to the pharmacist for manual review (Figure 2).

**FIGURE 3:**
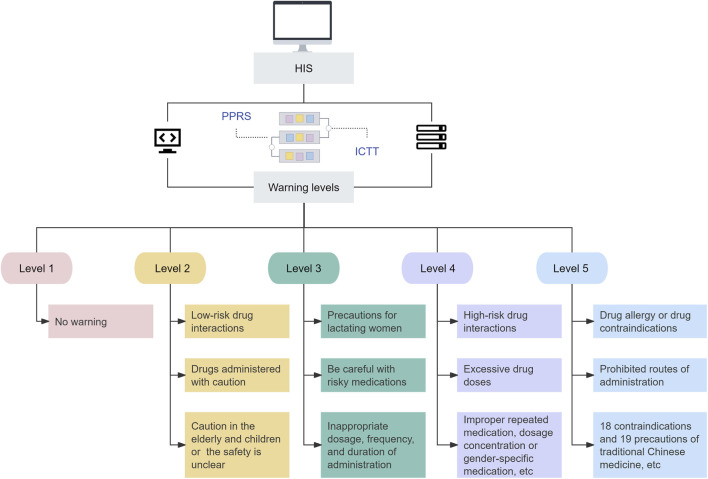
The levels and contents of drug warning information based on the ICTT within the PPRS. HIS: Hospital information system; PPRS: Pre-prescription review system; ICTT: Intelligent core think tank.

During this process, the PPRS generates alerts of different levels based on the severity of the problem in the prescription or medical order information transmitted by HIS (Figure 3). Among them, warnings at levels 2 to 5 will pop up notifications on the doctor’s workstation, while the warning at level 1 will only display background information in the background interface. Doctors can flexibly adopt appropriate actions based on the warning level and modification suggestions, including returning the original prescription for revision, deleting and reissuing the prescription, or submitting it to the pharmacist for review after providing the reason for medication. If approved by the pharmacist, the prescription can be saved and issued directly. Otherwise it will be returned for modification. If there are special reasons for medication, the doctor can explain the situation to the pharmacist. After assessment, the pharmacist will decide whether to approve the prescription. If it is not approved, the prescription will be returned to the doctor for reissuance (Figure 1).

### 3.2 Developing and maintaining the PPRS rule library

PPRS has established over 2.6 million foundational review rules based on ICTT (Supplementary Figure S1), covering the vast majority of drug usage rules in our hospital. Additionally, based on hospital medication needs and refined management methods, 7043 custom review rules were developed (Table 1), including hospital regulations (departmental, disease, and patient medication restrictions) and the developed rules (usage and dosage, route of administration, age and gender-specific medication, etc.) in the process of PPRS. The implementation and maintenance of these rules have standardized medication workflows, significantly reduced the error rate of prescriptions, and ensured patient medication safety. Through refined management pathways, we established PPRS rules that link patients' examination indicators to meet the individual medication needs, reduce inappropriate drug use, and thereby lower the incidence of adverse drug events. We will continue to optimize and update the system rules, and further standardize the medication process through refined management pathways to provide better services to safeguard patients' health.

**TABLE 1:** Custom review rules.

PPRS Rule Bank			
Custom rules	Drug interactions	Incompatibility contraindications	Administration route
	654	56	831
	Usage and dosage	Age restriction	Medication for the elderly
	3896	108	14
	Gender-specific medication	Medication during lactation	Medication during pregnancy
	5	4	5
	Adverse reactions	Drug contraindications	Compound concentration
	22	40	36
	Repeated medication	Important notice	Combination of antibiotics
	543	11	1
	Combination of Chinese patent medicines	Departmental limitations	Physician limitation
	73	11	6
	Patient limitation	Disease limitation	​
	12	715	​
Total	7043		

PPRS: Pre-prescription review system.

### 3.3 Refined management pathway of PPRS

#### 3.3.1 Indications setting and review of big data linkage

**FIGURE 4:**
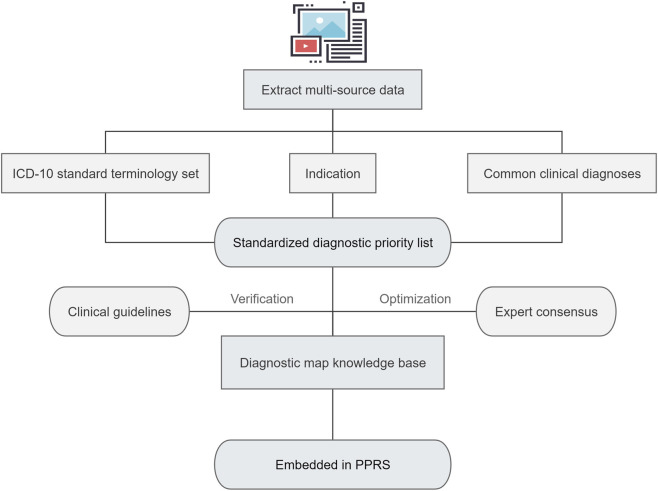
Flowchart for the construction of the diagnostic map knowledge base. PPRS: Pre-prescription review system.

Indication review is a key step to ensure drug safety. The precise matching degree between indications and drugs directly determines the efficiency and accuracy of review. Matching indications based on single drug package inserts is prone to false positives or missed reviews due to the diverse diagnostic terms used in clinical practice. Therefore, constructing a multi-resource integrated indication map is crucial. Our hospital has constructed a diagnostic map knowledge base based on the big data-driven review model (Figure 4). By linking HIS, ICTT and PPRS through data interfaces, we quantitatively matched the hospital’s ICD-10 standard term set, commonly used clinical diagnoses, and drug package inserts. Redundant information was removed, and results were ranked by matching accuracy to generate a prioritized list. Different weights were assigned, and the list was validated and optimized using guidelines and consensus documents before being embedded into PPRS. When the doctor issues prescriptions, the system automatically extracts diagnostic information and matches it with the diagnostic map knowledge base. High-weight (completely consistent diagnostic terms) or medium-weight (diagnoses closely related clinically but with differences in expression) are given priority for initial review, while low-weight (weak correlation) trigger manual intervention by pharmacists. This effectively improves the hit rate of diagnostic keywords, and the efficiency and accuracy of the review have been improved.

#### 3.3.2 Flexible flowchart customization and refined management module

In clinical practice, the complex medication requirements require that the PPRS has highly flexible custom functions to adapt the medication differences of different disease and populations. Refined management of PPRS can better meet the personalized needs of clinical medicine and ensure medication safety (Supplementary Figure S2). Based on flowchart design function, the system can linkage examination indicators according to the characteristics of the patient’s disease treatment to achieve the integration of “diagnosis-index-medication”, and provide risk stratification alerts for patients to ensure personalized medication needs. For example, human albumin injection is restricted by serum albumin levels when used for emergency treatment, critical conditions, or ascites/pleural effusion caused by liver cirrhosis or cancer (Supplementary Figure S3).

### 3.4 PPRS intervention outcomes

#### 3.4.1 Rationality rate of prescriptions and medical orders

**FIGURE 5:**
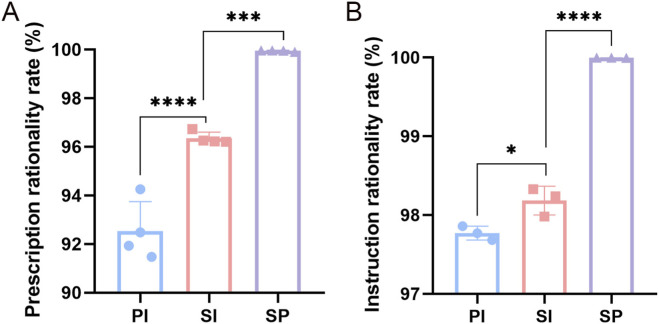
Rationality rates of prescriptions and medical orders before and after the PPRS intervention. **(A)** Changes in prescription rationality rate. **(B)** Changes in medical order rationality rate. PI: Pre-intervention; SI: System intervention; SP: System and pharmacist intervention. *P < 0.05; **P < 0.01; ***P < 0.001; ****P < 0.0001.

By collecting the data of outpatient prescriptions from 2021 to 2024 and inpatient medical orders from 2022 to 2024, we compared the rationality rates of prescriptions and medical orders before and after PPRS intervention. It was found that the rationality rates of both prescriptions and medical orders improved after the intervention (Figure 5). From 2021 to 2024, the rationality rate of total prescriptions increased from 92.53% to 96.35% following system intervention. After dual review by the system and pharmacists, the rationality rate further rose to 99.94% (Figure 5A). Similarly, the rate of reasonable medical orders also showed a significant increase. Since the launch and stable operation of the medical order review module in 2022, the total medical order rationality rate has risen from 97.77% to 99.99% (Figure 5B). Following the implementation of PPRS, the dual-review model involving both the system and pharmacists has significantly improved the rationality rate of prescriptions and medical orders. Furthermore, the residual irrationality in prescriptions and medical orders has decreased year by year (Supplementary Figure S4), enhancing the safety of clinical medication and ensuring the effectiveness and rationality of drug treatment.

#### 3.4.2 Proportion of unreasonable prescriptions types

**FIGURE 6:**
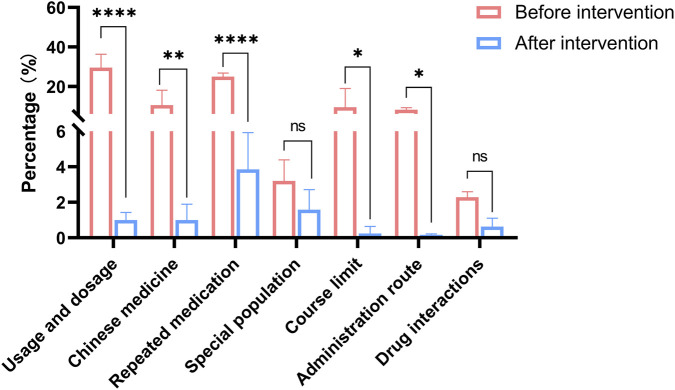
Rationality rate of unreasonable types of prescriptions in PPRS before and after intervention. *P < 0.05; **P < 0.01; ***P < 0.001; ****P < 0.0001; ns: non-significant.

After the system went online, data on the types of rules triggered before and after intervention were collected for analysis (Supplementary Table S1). From 2021 to 2024, the main problem prescription types in outpatient prescriptions were usage and dosage, repeated medication, and the combinations of Chinese patent medicines. For instance, the number of prescriptions with usage and dosage issues before the intervention in 2021 was 34,502, accounting for 34.31% of all problem prescriptions that year. After the intervention, it dropped to 1.30%. Notably, in 2024, the proportion of prescriptions with usage and dosage issues decreased to 19.51% before the intervention and further decreased to 0.77% after the intervention. This change can be attributed to the PPRS intervention standardizing doctors' prescription writing and pharmacists maintaining and updating the medication rules base. In addition, the proportion of prescriptions with repeated medication or combinations of Chinese patent medicines also changed significantly after RRPS intervention, dropping from 24.94% to 3.85% and from 13.99% to 1.33% respectively. Although, no statistically significant difference was observed in the problem prescriptions of special populations and drug interactions after PPRS intervention, the average proportion of these types of problem prescriptions also decreased by different degrees, accounting for 1.59% and 0.63% respectively after the intervention (Figure 6).

#### 3.4.3 Warning level change

The number of PPRS intercepted alerts has been increasing annually. Prescription alerts rose from 115,452 in 2021–196,179 in 2024 (Table 2), while medical order alerts increased from 74,861 in 2022–81,599 (Supplementary Table S2). Among them, Level 4 alerts accounted for the highest proportion, ranging from 71.52% to 78.60% in outpatient prescriptions and 42.40%–55.48% in inpatient medical orders. Level 3 and Level 5 followed, with prescriptions and medical orders accounting for approximately 10% and 20% of the annual total respectively. The number of Level 3 alerts in outpatient prescriptions (14,746–30,817) and their proportion (12.77%–15.71%) both showed a year-on-year increase. Level 2 alerts rose to 6.36% in the first 2 years after PPRS implementation and then dropped rapidly by 0.91%. However, inpatient medical order alerts for Level 2 (2.12%–0.91%) and Level 5 (32.55%–20.64%) decreased, while Level 4 alerts (42.40%–55.48%) increased annually (Table 2; Supplementary Table S2). The changes in the number and proportion of these alerts indicate that through the continuous optimization and refinement of the system by pharmacists, using different alert levels can not only enhance doctors' attention to prescription standards, but also reduce the occurrence of ineffective alerts, so as to better serve the clinic practice, reduce the incidence of adverse drug events, and promote rational drug use among patients.

**TABLE 2:** Number and proportion of PPRS alert levels triggered by outpatient prescriptions (from 2021 to 2024).

Year	Level 2		Level 3		Level 4		Level 5		Total	
	N	%	N	%	N	%	N	%	N	%
2021	3014	2.61	14,746	12.77	90,746	78.60	6946	6.02	115,452	100
2022	7639	6.36	15,659	13.05	85,858	71.52	10,888	9.07	120,044	100
2023	4107	3.01	20,688	15.17	100,707	73.83	10,901	7.99	136,403	100
2024	1787	0.91	30,817	15.71	144,108	73.46	19,467	9.92	196,179	100

PPRS: Pre-prescription review system.

## 4 Discussion

PPRS, as a core component of medication management processes, has redefined drug safety practices through real-time proactive intervention in advance, demonstrating its unique ability to reduce preventable adverse drug events (ADEs) in outpatient and inpatient Settings (Carollo et al., 2024; Yang et al., 2025; Yue et al., 2025), has become an essential tool for standardizing physicians' prescribing behaviors, mitigating medication errors, and facilitating closed-loop management throughout the entire medication process. In the high-throughput pharmacy environment with huge human flow, PPRS enables pharmacists to prioritize high-severity alerts and provide more rapid medication guidance and feedback through alert severity grading (Xie et al., 2022; Guo et al., 2025; Yang et al., 2025), which improves the efficiency of prescription review.

This study retrospectively analyzed the potential impact of the dual review model combining PPRS and pharmacist manual review on the rationality of outpatient prescriptions and inpatient orders over the past 4 years since PPRS was launched. As an auxiliary tool for pharmacists to review prescriptions, PPRS has significantly improved the rationality of outpatient prescriptions and inpatient orders. Under the dual intervention mode of PPRS and pharmacists, the pass rate has reached approximately 99.9% (Figure 4). In addition, PPRS can reduce medication errors by sending different levels and types of drug warning information to doctors (Table 2; Supplementary Tables S1, 2). Among these alerts, alerts related to usage and dosage (excessive/insufficient doses, incorrect administration frequency) are the most common cause of medication errors (Aronson, 2009). Setting alerts for abnormal usage and dosage in PPRS is essential to reduce adverse drug events caused by doctors' medication errors (Jiménez Muñioz et al., 2010; Gates et al., 2021).

Similar to previous studies (Xie et al., 2022; Yue et al., 2025), this research found that PPRS has provided a relatively significant benefit in reducing the rate of irrational drug use, which is helpful to standardize the clinical practice of prescription and medical orders. Notably, in this study, medication errors were more prevalent in outpatient prescriptions at hospitals. The total number of alerts in 2024 has reached 196,179, while the highest number of inpatient medical order alerts was 83,643 in 2023 (Table 2; Supplementary Table S2). This may be attributed to the important role of clinical pharmacists in the clinical practice process and their contribution in assisting doctors in managing patient treatment and ensuring rational drug use in clinical practice. In addition, the well-developed full process management system is commendable. Previous studies mainly focused on evaluating the standalone effectiveness of PPRS after implementation (Liu et al., 2021; Yang et al., 2025), yet they demonstrate limited adaptability to complex clinical scenarios. yet they demonstrate limited adaptability to complex clinical scenarios. Similarly, international mainstream clinical decision support systems (CDSS) face common challenges, including insufficient precision and unacceptably high false-positive rates (Tsai et al., 2016; Sutton et al., 2020). In contrast, our PPRS integrates institutional medication management protocols and professional clinical guidelines to standardize doctors' prescription behavior enhance therapeutic appropriateness, and ensure the regulated supply of specialized medications. Meanwhile, PPRS is also crucial in meeting the individual medication needs of patients. For special groups (high risk of drug-related harm) and patients with abnormal laboratory test results, PPRS provides risk stratification to patients by cross-referencing package inserts, drug administration guidelines for specific populations, and linking examination indicators.

However, it should be acknowledged that some limitations of the current RPRS still exist. Firstly, false positive alerts and system omissions remain unavoidable, and delays persist in transmitting dynamic data for specific patients between HIS and PPRS, thereby limiting the PPRS’s early warning capabilities. Secondly, after drug replacements, there may be a lag in the update of package inserts, guidelines and disease-specific knowledge bases in the ICTT database, and some reasonable prescriptions may be wrongly attributed to unreasonable use. What’s more, the existing review rules with the weak ability of fine adaptation and dynamic adjustment of the original underlying database may not be able to fully identify potential irrational drug use in complex situations, and the system standardized review rules are insufficiently adapted to clinical diagnosis and treatment (clinical individual drug needs), which poses new challenges for the subsequent work of the prescription review pharmacists. Although the rationality rates of prescriptions and medical orders have both improved after PPRS intervention, the intervention of pharmacists is still indispensable. Particularly in complex medication situations, precise medication control requires pharmacists to make reasonable judgments based on clinical practice. Finally, PPRS requires continuous improvement and advancement through refined management approaches to meet the clinical medication needs, which depends on the maintenance and update of PPRS by pharmacists and their deep and comprehensive understanding of rational drug use. Therefore, the dual review model of PPRS and pharmacists remains one of the most important approaches to ensure rational drug use.

This study demonstrates that the implementation of PPRS has shown significant value in promoting rational drug use and reducing adverse reactions, providing a replicable template for smart medical care to assist clinical practice. PPRS has been deployed and operated stably in two other large tertiary centers in Chongqing, and will be extended to regional medical alliances in the next step. The regional centralized review center model of the medical consortium would be a good solution. By comprehensively considering the actual needs of grassroots hospitals, the basic review of PPRS can be given priority, and regular training and assistance can be provided. Subsequently, it can be iterated step by step and managed in a more refined manner. In the future, by integrating machine learning to determine unified integration indicators and combining with an intelligent platform to regularly update and automatically upgrade the PPRS (Sahni and Carrus, 2023), it will assist pharmacists in further reducing false positives, errors or ineffective alerts, and play an important role in continuously optimizing PPRS rules and promoting more comprehensive rational clinical drug use.

## 5 Conclusion

In summary, the implementation and refined management of PPRS at a large tertiary general hospital in western China have effectively verified that the dual review model combining of PPRS combined with manual review by pharmacists is significantly associated with improved prescriptions and medical orders rationality, thereby reducing the damage caused by drug-related issues and ensuring the safety of medication. The real-world application and refined management path of PPRS provided by this study offers a reproducible template for the implementation of smart assistance in clinical practice, which is of great significance for the development and construction of global digital health practice and smart hospitals.

## Data Availability

The original contributions presented in the study are included in the article/Supplementary Material, further inquiries can be directed to the corresponding authors.
