# Supplementary material for: Construction and refined management of a pre-prescription review system: a real-world study in a tertiary hospital
Source: Front Pharmacol. 2026 Mar 26;17:1791155. doi: 10.3389/fphar.2026.1791155 (PMC13062318; doi:10.3389/fphar.2026.1791155)
Supplement: Supplementary file 1 [file Supplementaryfile1.docx]

***Supplementary Material***


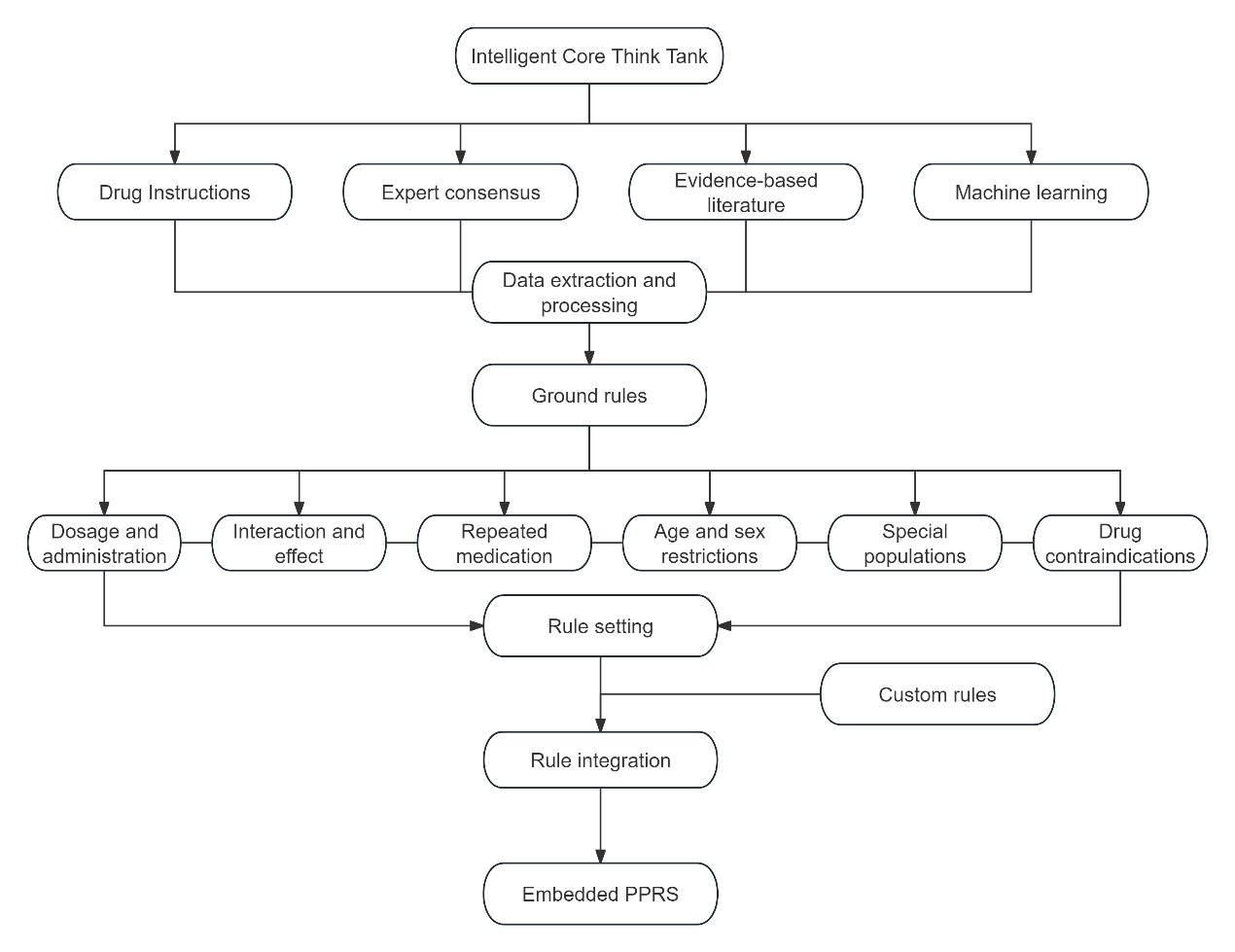


Figure S1. Flowchart for building the intelligent core think tank (ICTT).


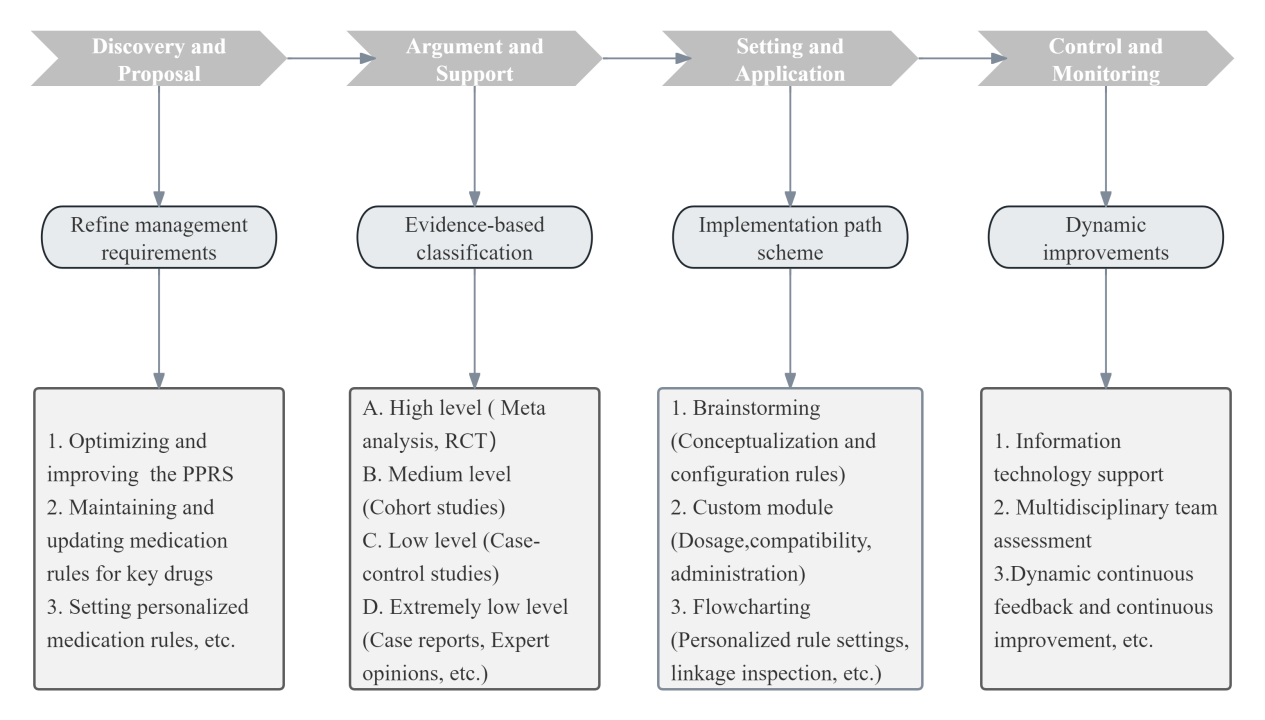


Figure S2. An overview of the refined management process of PPRS. PPRS: Pre-prescription review system; RCT: Randomized controlled trial.


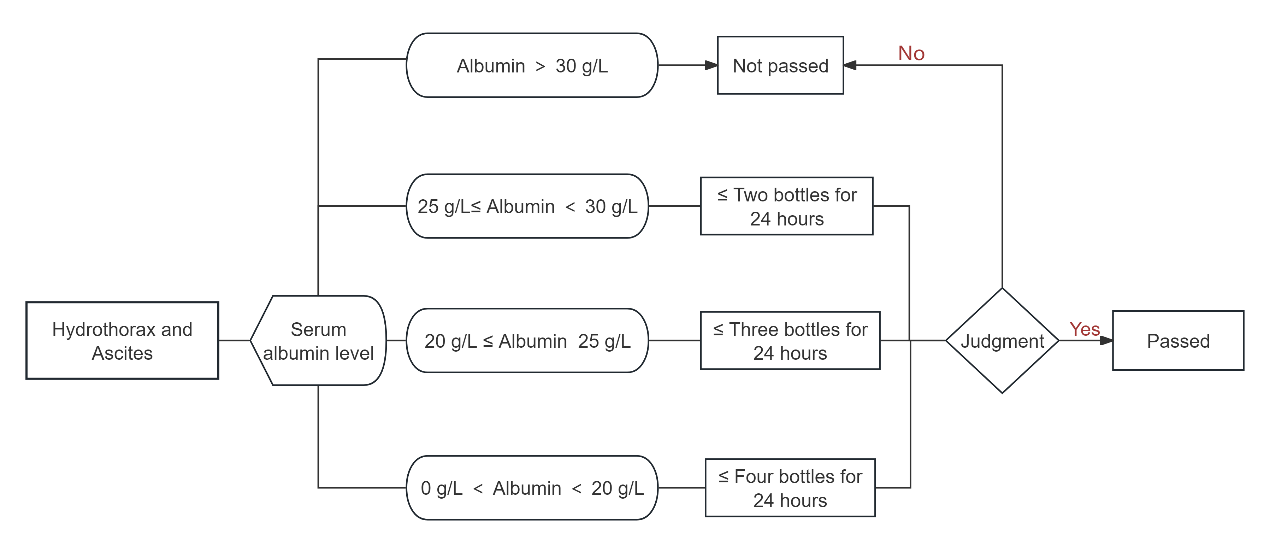


Figure S3. Flowchart example of the rules for the use of human albumin injection.


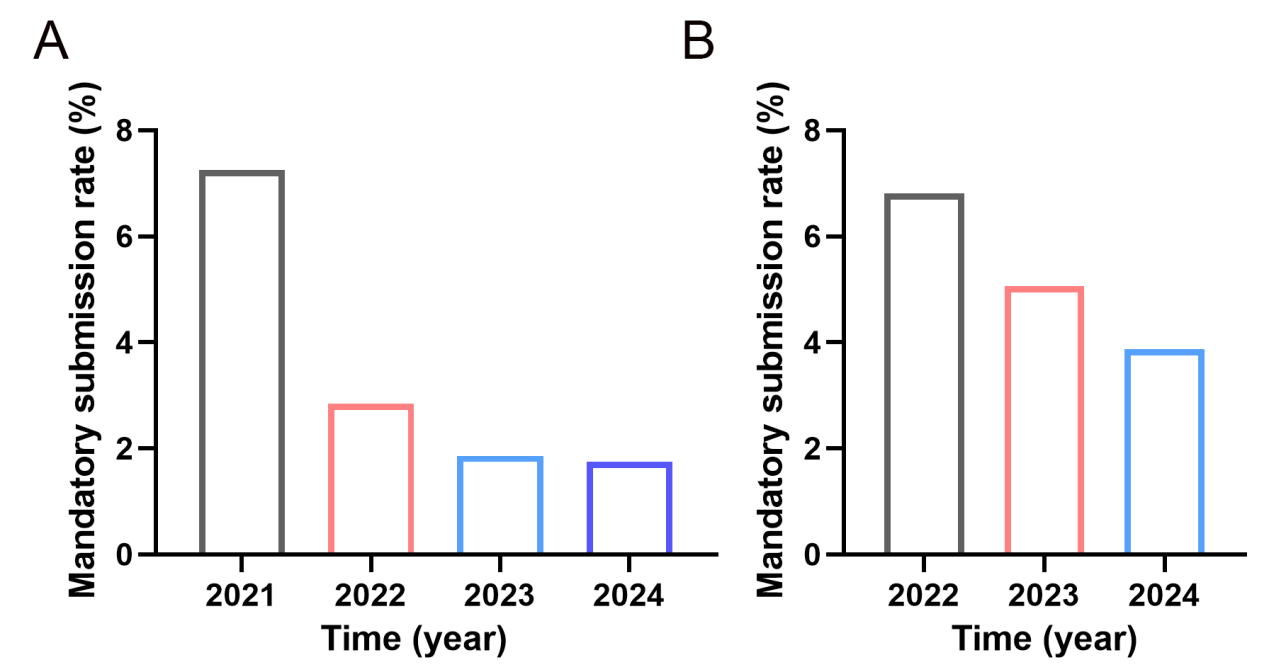


Figure S4. Residual irrationality in prescriptions and medical orders after intervention. (A) Prescription residual irrational rate; (B) Medical order residual irrationality rate.

Table S1. Number of PPRS alert types triggered before and after the intervention (from 2021 to 2024)

| **Alert types** | **Year** | | | | | | | |
| --- | --- | --- | --- | --- | --- | --- | --- | --- |
|  | **2021** | **2022** | **2023** | **2024** | **2021** | **2022** | **2023** | **2024** |
|  | **Before intervention** | | | | **After intervention** | | | |
| Usage and Dosage | 34502 | 32394 | 38633 | 32775 | 1310 | 545 | 1647 | 1295 |
| Chinese Medicine | 14071 | 12375 | 21044 | 20308 | 1341 | 661 | 2431 | 2149 |
| Repeated medication | 23990 | 27856 | 30760 | 38521 | 2477 | 661 | 4473 | 3885 |
| Special population | 2477 | 1647 | 4473 | 3885 | 661 | 2374 | 695 | 266 |
| Course limit | 5288 | 4589 | 5274 | 23885 | 61 | 49 | 43 | 1415 |
| Administration route | 9493 | 8391 | 9853 | 11690 | 229 | 134 | 154 | 318 |
| Drug interactions | 2346 | 2840 | 2468 | 3330 | 329 | 1320 | 876 | 312 |

PPRS: Pre-prescription review system.

Table S2. Number and proportion of PPRS alert levels triggered by inpatient medical orders (from 2022 to 2024)

| **Year** | **Level 2** | | **Level 3** | | **Level 4** | | **Level 5** | | **Total** | |
| --- | --- | --- | --- | --- | --- | --- | --- | --- | --- | --- |
|  | **N** | **%** | **N** | **%** | **N** | **%** | **N** | **%** | **N** | **%** |
| 2022 | 1586 | 2.12 | 17165 | 22.93 | 31744 | 42.40 | 24366 | 32.55 | 74861 | 100 |
| 2023 | 1643 | 1.96 | 18646 | 22.29 | 44628 | 53.35 | 18726 | 22.40 | 83643 | 100 |
| 2024 | 848 | 1.04 | 18634 | 22.84 | 45275 | 55.48 | 16842 | 20.64 | 81599 | 100 |

PPRS: Pre-prescription review system.
