# Supplementary material for: Construction and refined management of a pre-prescription review system: a real-world study in a tertiary hospital
Source: Front Pharmacol. 2026 Mar 26;17:1791155. doi: 10.3389/fphar.2026.1791155 (PMC13062318; doi:10.3389/fphar.2026.1791155)
Supplement: Supplementary file 2 [file Supplementaryfile2.docx]

**Supplemental Digital Content, Table.** Final EBPQI Reporting Guideline with Example

| **Component** | **Description** | **Example** |
| --- | --- | --- |
| **1. ABSTRACT** | The abstract summarizes the main points of the manuscript, including a statement that an EBPQI initiative was conducted. | This study conducted a retrospective analysis of the establishment of the pre-prescription review system (PPRS) and its effectiveness in promoting rational drug use, and further explored pathways for its refined management. |
| **2. INTRODUCTION** | Identifies the problem to be addressed by the EBPQI initiative. | With the support of the information technology-driven PPRS, how to strike a balance between review efficiency and accuracy? |
| **3. Background** | Includes a description of the general and local practice problem with supporting evidence (eg, epidemiological, economic, health). | General problem: existing PPRS is limited in applicability in complex clinical scenarios, suffering from insufficient accuracy and high false-positive rates.  Local problem: in high-throughput pharmacy environments, effective pharmacist interventions require more refined PPRS to achieve a balance between review efficiency and accuracy. |
| **4.** **External evidence review and evidence-based practice components** | | |
| ***4.1 Evidence search strategy*** | Includes an evidence searching question (PPCO, PICO), key terms, and databases searched. Does not require a PRISMA flowchart. | Not applicable. This study is a single-center real-world retrospective study and does not involve database searches. |
| ***4.2 Critical appraisal and evidence synthesis*** | Includes a narrative synthesis of the *quality* of the evidence based on findings from critical appraisal tools (eg, Critical Appraisal Skills Program checklists, Center for Evidence-Based Medicine critical appraisal tools, Johns Hopkins critical appraisal tools).  Also includes a narrative synthesis of the *evidence* that will be used for the EBP recommendation. The best quality and most recent evidence should be included. | Before and after the PPRS went online, prescription and inpatient order data were monitored. Unpaired sample t-test and one-way ANOVA were used to study the primary outcome of the rationality rate of prescriptions and medical orders, and the secondary outcome of the types of unreasonable prescriptions and the changes in system warning levels. Using evidence-based methods, a descriptive analysis was conducted on the construction and refined management path of PPRS, and its effectiveness was evaluated. The results indicated that the construction and refined management of the PPRS can help improve the rationality of prescriptions and medical orders, promote personalized medication, and establish a replicable model for the clinical practice of smart healthcare. |
| ***4.3 Clinical expertise*** | Includes a description of the clinical expertise that informed the EBP recommendation (eg, what experts were included on the EBPQI team that helped identify the EBP recommendation?). | A multidisciplinary team (pharmacy department, clinical departments, information department, etc.) was established to communicate and cooperate with the system architecture engineers (Sichuan Youlian) to accurately identify clinical needs, jointly design the PPRS, and conduct refined management during its subsequent operation, so as to adapt to complex clinical scenarios and better balance the efficiency and accuracy of the review process. |
| ***4.4 Patient/ Population values/preference/ culture*** | Includes a description of patient/population values, preferences, and culture were considered when developing the EBP recommendation. | Not applicable. The construction and refinement management of PPRS mainly lie in assisting pharmacists in reviewing, standardizing doctors' behaviors, and promoting rational drug use. Therefore, members of the multidisciplinary team include those from the pharmacy department, clinical department, information department, etc. |
| **5. Internal Evidence** | Internal evidence is included (may be in the introduction or background) to document that there is a local problem and may be used as the baseline for improvement measures. | Internal evidence shows that in high-throughput pharmacy environment, pharmacists review a large number of prescriptions daily, with the quantity possibly exceeding 10000. On average, they need to complete the review of one prescription every 30 seconds. In this high-intensity working condition, how to balance the review efficiency and accuracy has become a critical issue. |
| **6. Purpose and Aims** | Includes a clear purpose statement and aims written using the SMART criteria (specific, measurable, achievable, relevant, time-bound). The aims should align with the appropriate outcome, balancing, and process measures. | This study aims to conduct a retrospective analysis of the application effects of PPRS in rational drug use and its refined management. The specific measurement indicators are as follows: (1) the construction and refined management of PPRS have significantly increased the rationality rate between the total prescriptions (92.53% *vs* 99.94%) and medical orders (97.77% *vs* 99.99%); (2) the proportion of prescriptions with high problem proportions decreased significantly after intervention, such as repeated medication (24.94% *vs* 3.85%) and incorrect usage and dosage (34.31% *vs* 19.51%). (3) the accuracy rate of PPRS has improved, and the number of alerts intercepted by PPRS has increased year by year, with a decrease in invalid alerts. For example, the proportion of Level 3 prescription alerts (12.77% *vs* 15.71%) and Level 4 medical orders alerts (42.40% *vs* 55.48%) has risen, while the proportion of Level 2 alerts for prescriptions (2.61% vs 0.91%) and medical orders (2.12% vs 1.04%) generally showed a downward trend. |
| **7. METHODS** | | |
| **7.1. Framework or Model Design** | A framework or model to guide the QI methods are described (eg, PDSA, DMAIC). Research methods (eg, quasi-experimental pre-post study, cross-sectional survey) are inappropriate. | The research methods (eg, PDSA, DMAIC) are not applicable. A single-center real-world retrospective analysis study was conducted. Using evidence-based methods,a descriptive analysis was conducted on the construction and refined management path of PPRS. Unpaired sample t-test and one-way ANOVA were used to study the primary outcome of the rationality rate of prescriptions and medical orders, and the secondary outcome of the types of unreasonable prescriptions and the changes in system warning levels. |
| **7.2 Context and population** | The local environment (context) where the practice change was implemented is described.  A description of the population to which the practice change applies to is included. This should not be listed as “patient sample” or include “inclusion/exclusion criteria” as these are research terms and are not applicable to EBPQI initiatives. | This study was conducted at Southwest Hospital, a comprehensive tertiary grade-A hospital in Chongqing, China. This study collected the data (pre-intervention data) monitored by the prescription automatic screening system (PASS) during the six months prior to the launch of the PPRS and the data of drug prescriptions and inpatient medical orders since the PPRS was implemented in November 2020. |
| **7.3 Engagement** | The EBPQI team members are described. | The EBPQI team included a multidisciplinary team (pharmacy department, clinical departments, information department, etc.) worked together with the system architecture engineer (Sichuan Youlian). |
| **7.4 Ethical Consideration** | Addresses ethical components of EBPQI work with disclosure of review/determination process. If appropriate, a statement about confidentiality may be included. | This study does not involve data from animals or humans, and has been reviewed and approved by the confidentiality committee of southwest hospital. |
| **7.5 Description of tests of practice change** | Includes a description of the iterative tests (eg, more than one assessment after a change is implemented) of practice change during the evaluation period. Changes should be implemented using evidence-based implementation strategies. | Based on the clinical practice requirements and the refined management pathway, the review rules were dynamically adjusted after the launch of PPRS. Practice changes were implemented using evidence-based strategies, including the assessment by a multidisciplinary team (pharmacy department, clinical departments, information department, etc.), the trial operation of the rules, and the dynamic adjustment. |
| **7.6 Measures and analysis** | For each specific aim, include the operational definition (eg, what data were collected, how data collection occurred). Include how data were analyzed using QI methods (ie, data are collected at regular intervals, plotted on a run chart or statistical process control chart, and continuously analyzed to determine effectiveness of the practice change).  EBPQI initiatives may use descriptive statistics; however, inferential statistics are usually inappropriate, and a power analysis is not required. | **Collected data：**this study collected the data (pre-intervention data) monitored by the prescription automatic screening system (PASS) during the six months prior to the launch of the PPRS and the data of drug prescriptions and inpatient medical orders since the PPRS was implemented in November 2020.  **Data collection methods:** the prescription and medical order data of 6 months before the launch of PPRS were retrieved in PASS, and the prescription and medical order data monitored since the launch of PPRS in November 2020 were retrieved by the PPRS, and the above data were imported into the excel sheet for record classification.  **Data analysis methods:**using evidence-based methods, a descriptive analysis was conducted on the construction and refined management path of PPRS. Unpaired sample t-test and one-way ANOVA were used to study the primary outcome of the rationality rate of prescriptions and medical orders, and the secondary outcome of the types of unreasonable prescriptions and the changes in system warning levels. |
| **8. RESULTS** | Results section addresses each stated specific aim. Includes data visualization (eg, run charts or statistical process control charts). | The results of this study showed: (1) PPRS has been constructed and managed in a refined manner, better aligning with clinical practice and facilitating personalized medication. Examples of this can be found in the manuscript and the illustrative sections of the supplementary materials. (2) the construction and refined management of PPRS have significantly increased the rationality rate between the total prescriptions (92.53% *vs* 99.94%) and medical orders (97.77% *vs* 99.99%); (3) the proportion of prescriptions with high problem proportions decreased significantly after intervention, such as repeated medication (24.94% *vs* 3.85%) and incorrect usage and dosage (34.31% *vs* 19.51%). (4) the accuracy rate of PPRS has improved, and the number of alerts intercepted by PPRS has increased year by year, with a decrease in invalid alerts. For example, the proportion of Level 3 prescription alerts (12.77% *vs* 15.71%) and Level 4 medical orders alerts (42.40% *vs* 55.48%) has risen, while the proportion of Level 2 alerts for prescriptions (2.61% vs 0.91%) and medical orders (2.12% vs 1.04%) generally showed a downward trend. |
| **9. DISCUSSION** | Discussion includes a summary of the results and comparison to the synthesis of previous evidence. The discussion section should also address initiative sustainability. | Overall, this research found that PPRS has provided a relatively significant benefit in reducing the rate of irrational drug use, which is helpful to standardize the clinical practice of prescription and medical orders (similar to previous studies). Moreover, under the guidance of the refined management path, our research has established a replicable template to support clinical practice in the field of intelligent healthcare, reduce ineffective alerts, and promote individualized drug use. We will continue to update and automatically upgrade PPRS in combination with the intelligent platform, continuously optimize PPRS rules, and promote more comprehensive clinical rational drug use. |
| **10. Limitations** | Limitations address unavoidable and unpredicted occurrences and lessons learned. | There is sometimes a delay in the dynamic data transmission of the system, false alarms and system omissions still cannot be avoided; when the drugs are replaced, the PPRS may have data update lag, thereby wrongly judging the rationality of the prescription; the existing review rules have weak adaptability and dynamic adjustment capabilities, and are unable to fully identify potential irrational drug use behaviors in complex situations. |
| **11. CONCLUSIONS** | Frame results and implications for future practice and continued improvement. | The real-world application and refined management path of PPRS provided by this study offers a reproducible template for the implementation of smart assistance in clinical practice, which is of great significance for the development and construction of global digital health practice and smart hospitals.In the future, practices can play an important role in identifying unified integration indicators by integrating machine learning step by step iterations and managing them in a more refined way, continuously optimizing PPRS rules and promoting more comprehensive clinical rational drug use |

EBPQI=evidence-based practice quality improvement; EBP=evidence-based practice; QI=quality improvement
